# Iodine in Continued Fever

**Published:** 1854-11

**Authors:** G. W. Hall

**Affiliations:** Carthage, Ills.


### IODINE IN CONTINUED FEVER, PHARYNGITIS AND PNEUMONIA.

BY DR. G. W. HALL, CARTHAGE, ILL.

I was induced to try the effects of Iodine in continued Fever, from seeing a statement in some journal that a physician in one of the Southern States had been using it in this affliction with success. I have forgotten the name and location of the physician, and can only make this general reference.

In the first case in which I tried it, I gave it on the seventh day of the fever, after the usual remedies generally employed in a case of continued fever without any discoverable local complication, and the *abortive* method had been insufficient to arrest the febrile symptoms. In two days more, during which the iodine was continued, the fever, according to common parlance, was "broken," and the patient speedily convalesced, without a relapse or any untoward symptom. The next case in which I gave it, I will give more in detail.

I was called on the 10th day of April last, to see a little girl of Mr. S., aged eight years. She was taken sick on the 6th, with a chill, followed by fever, which had continued until the time I was called, without any discernible remission, so far as I could learn. Diarrhœa had set in two days before I was called. She had delirium at night, caused perhaps by a loosing exacerbation. Pulse 120, small and weak; skin hot and dry. Tenderness on pressure in the right iliac region. Tongue dry and covered with a brownish coating. No eruption. I prescribed calomel and ipicacuanha; applied a blister over the abdomen, and directed her attendants to sponge her frequently in cold water, and to apply cloths wet in cold water to her head.

Next morning there was an admixture of bile in the discharges from the bowels—tongue a little moist. Same in other respects. Five grains of quinine were ordered every three hours. No better in the evening. A mixture of turpentine and laudanum was prepared and several doses given through the night. At the morning visit next day I found her weaker; sordes were collecting on the teeth; tongue dry and fissured; diarrhœa undiminished; blister drying. At this visit I dissolved two grains of iodine, with six grains of iodide of potassium, in an ounce of water, and directed her mother to give fifteen drops every two hours. In the evening her tongue was again a little moist; discharges from the bowels not so frequent. She had no delirium that night. Next morning the tongue was cleaning, and the skin, which had previously been dry, was now moist. From this time she quickly recovered; the iodine was continued two days longer, at which time she was discharged from treatment.

I have since used it in *four* similar cases, and the same good results have ensued. I must say however that I doubt its always being attended with the same results. Other cases *apparently* similar to the above may be in more respects than one, widely different. And then owing often to some inscrutable cause, such is the diversity in the action of remedies, that we can rely with entire certainty on the powers of none. Still, I believe that in cases *precisely similar* to the above, the iodine would have the same effect. If others should be induced to try it, I trust they will give the profession the result of their experience. In the Boston Medical and Surgical Journal of November 16th, 1853, there is an article from Dr. A. P. Merrill, of Nashville, Tenn., in which he recommends the iodine as a local remedy of value in pharyngitis.

Since I saw that I have treated several cases with it, and I believe it acts as well and often better than the nitrate of silver. In acute tonsilitis it will be found, I think, to be more prompt in arresting the inflammatory process, than almost any other remedy.

Dr. Merrill's formula is as follows:

| | | |
|---|---|---|
| ℞ Iod. Potass. | | ʒj. |
| Iodine, | | ʒss. |
| Sugar, and Gum Arabic, | *aa* | ʒij. |
| Water, | | ℥j. |

M. To be applied to the part affected with a camel's hair pencil. If too strong, to be diluted by adding warm water.

In the last two years I have used the iodine and iodide of potass., in many cases of pneumonia, and the cases in which it was given have recovered sooner, and done better than under any other treatment I have ever tried. How it acts I do not know; but certain it is that in my hands it has tended powerfully and promptly towards producing an amelioration of the symptoms and a resolution of the inflammation.

It relieves in a very favorable manner, the distressing cough, dispnœa, and heat of surface.

Carthage, Ills. July 22, 1854.

